# Lithium in severe affective disorders: Balancing safety with efficacy

**DOI:** 10.1192/j.eurpsy.2021.2060

**Published:** 2021-08-13

**Authors:** R. Almeida Leite, M. Almeida, J. Borges, A. Costa

**Affiliations:** Psychiatry And Mental Health, Baixo Vouga Hospital Centre, Aveiro, Portugal

**Keywords:** lithium, bipolar affective disorder, Suicide, side effects

## Abstract

**Introduction:**

Lithium has been one of the oldest substances used in psychiatric treatments and remains the first-line treatment for prevention of manic and depressive episodes of bipolar disorder (BD), but it has also a wide spectrum of side-effects.

**Objectives:**

The goal is to review efficacy, and clinical use of lithium, such as its side effects, and its benefit-to-risk ratio.

**Methods:**

Non-systematic literature review based on scientific databases such as PubMed.

**Results:**

The first modern use of lithium was for the treatment of mania. Lithium has also proven useful in major depression, particularly for augmentation of antidepressants, for aggressive behavior and it has a specific antisuicide effect. Lithium’s prophylactic and antisuicidal effects are most unique. However, the use of lithium became problematic due to the serious toxicity since lithium also a narrow therapeutic index, with therapeutic levels between 0.6 and 1.5 mEq/L.

**Conclusions:**

Awareness of the benefits and risks of lithium is essential for the use of this lifesaving agent. Lithium levels must be carefully monitored and lithium dosage adjusted as necessary.

**Disclosure:**

No significant relationships.

